# Autoencoders for unsupervised analysis of rat myeloarchitecture

**DOI:** 10.1007/s00429-026-03166-w

**Published:** 2026-09-26

**Authors:** Melina Estela, Raimo A. Salo, Isabel San Martín Molina, Omar Narvaez, Ville Kolehmainen, Jussi Tohka, Alejandra Sierra

**Affiliations:** 1 A.I. Virtanen Institute for Molecular Sciences, University of Eastern Finland, Kuopio, Finland; 2 Department of Applied Physics, University of Eastern Finland, Kuopio, Finland

**Keywords:** Histological image analysis, Convolutional autoencoders, PCA, DINOv3, Tissue clustering, Myelin, Traumatic brain injury, Computational pathology

## Abstract

Quantitative assessment of brain histology is often constrained by predefined feature sets and labor-intensive manual annotations. To overcome these limitations, we employed unsupervised deep learning to automatically extract and quantify tissue organizational patterns from myelin-stained rat brain sections without the need for prior labeling. We evaluated nonlinear convolutional autoencoders (AEs) against linear principal component analysis (PCA) and DINOv3, a self-supervised foundation model applied without task-specific fine-tuning, for feature representation, followed by clustering with Gaussian mixture models. Compared to both PCA and DINOv3, AEs better preserved fine axonal architecture and produced more consistent and interpretable tissue clusters across hierarchical levels. The resulting clusters revealed anatomically meaningful tissue organization, including different white matter densities and grey matter subregions. When applied to tissue from sham and mild traumatic brain injury animals, AE-derived features also captured pathology-related alterations, such as white matter loss and injury-specific microstructural changes. These findings suggest that task-specific unsupervised training on domain-specific histological data can automatically characterize tissue organization at multiple scales and detect pathological changes, offering a scalable, annotation-free approach to computational neuropathology.

**Supplementary Information:**

The online version contains supplementary material available at https://doi.org/10.1007/s00429-026-03166-w.

## Introduction

Histological analysis of healthy and diseased brain tissue has traditionally relied on expert visual inspection of stained sections to assess tissue architecture and identify pathological patterns. Over the last decades, automated whole-slide scanners have begun transforming histology (Al-Janabi et al. 2012; Gurcan et al. 2009; Webster and Dunstan 2014; Wu et al. 2022) by enabling high-resolution digitization of large numbers of tissue sections. While this development has increased data volume and quality, it has also increased the workload of neuropathologists and neuroscientists. Consequently, the growth in digital histology data has created both an opportunity and a necessity for computational methods (Amunts et al. 2022; Gurcan et al. 2009), marking a transition from purely qualitative visual assessment toward quantitative, algorithm-driven analysis.

A major limitation of current quantitative histology workflows is their dependence on visual morphological assessment (Elmore et al. 2017; Schmitz and Hof 2005) and manual delineation of regions of interest. Although effective for targeted analyses, these approaches are time-consuming, subjective, and difficult to scale to whole-section or whole-brain datasets. To address these challenges, several open-source software, such as QuPath (Bankhead et al. 2017), Fiji (Schindelin et al. 2012), and others (Piccinini et al. 2025; Zuraw and Aeffner 2022), automate parts of the image analysis pipeline, including cell detection, staining-intensity quantification, and morphometric measurements across large tissue areas. However, these tools remain largely object-centric, relying on predefined features, parameter tuning, or manual input, yet they do not provide whole characterization of brain tissue sections (Webster and Dunstan 2014).

In this context, computational histopathology has advanced substantially through supervised deep learning models trained on large annotated datasets (Huang et al. 2025; Wu et al. 2022). These models enable automated prognosis prediction (Cooper et al. 2023; Huang et al. 2025), segmentation (Cooper et al. 2023; Schiffer et al. 2021), and tumor classification (Aggarwal et al. 2025; Campanella et al. 2019; Ding et al. 2025; Graham et al. 2023; Hou et al. 2016; Ker et al. 2019; Su et al. 2022). Similar approaches can be applied to brain tissue, supporting cell detection (Vadori et al. 2024), segmentation of cellular and subcellular structures, cytoarchitectonic mapping (Schiffer et al. 2021), and myelin analysis (McKenzie et al. 2022). However, these supervised approaches remain constrained by their need for annotated training data and predetermined tissue classes, limiting their ability to capture subtle variations in brain tissue architecture (Zuraw and Aeffner 2022).

Unsupervised learning offers an alternative for discovering patterns directly from data without manual annotations. While unsupervised deep learning has been applied in non-brain tissues (Rmouque et al. 2022; Rumman et al. 2023; Sheikh et al. 2022), it has not yet been applied systematically to myelin-stained brain sections, where axonal orientations, fiber crossings, and cellular distributions form complex microstructural patterns. A central question in applying unsupervised methods to histological data is whether nonlinear feature-extraction techniques provide meaningful advantages over classical linear approaches. Nonlinear methods, such as autoencoders (AEs) (Goodfellow et al. 2016), can capture complex biological structure but require more computational resources, while linear methods, such as principal component analysis (PCA) offer interpretability and efficiency. Recent advances in self-supervised learning have also further expanded the landscape of feature extraction, with vision foundation models such as DINO (Siméoni et al. 2025) learning rich visual representations from large-scale image data. Although domain-specific fine-tuning of such models has been explored in histopathology (Chen et al. 2024; Saillard et al. 2024; Zimmermann et al. 2024), the training datasets of these models rarely include specialised staining protocols such as gold-chloride stain for myelin, which may limit their out-of-the-box applicability to myeloarchitectural analysis.

In this work, we applied convolutional AEs to extract features from myelin-stained rat brain sections and used unsupervised clustering to identify distinct patterns of myeloarchitecture, addressing the question of whether complex myelin organization can be resolved and characterized without manual annotation or predefined tissue classes. To contextualize our approach, we compared AE-derived representations against PCA, as a classical linear baseline, and DINOv3 (Siméoni et al. 2025), a self-supervised foundation model applied without task-specific fine-tuning. This comparison allows us to assess whether training directly on myelin-stained patches offers advantages over both classical decomposition and generic visual representations for capturing the fine-grained complexity of axon density, orientations, and fiber crossings that characterize myeloarchitecture.

## Results

### Unsupervised clustering workflow results in systematic myelin tissue classification

**Fig. 1:**
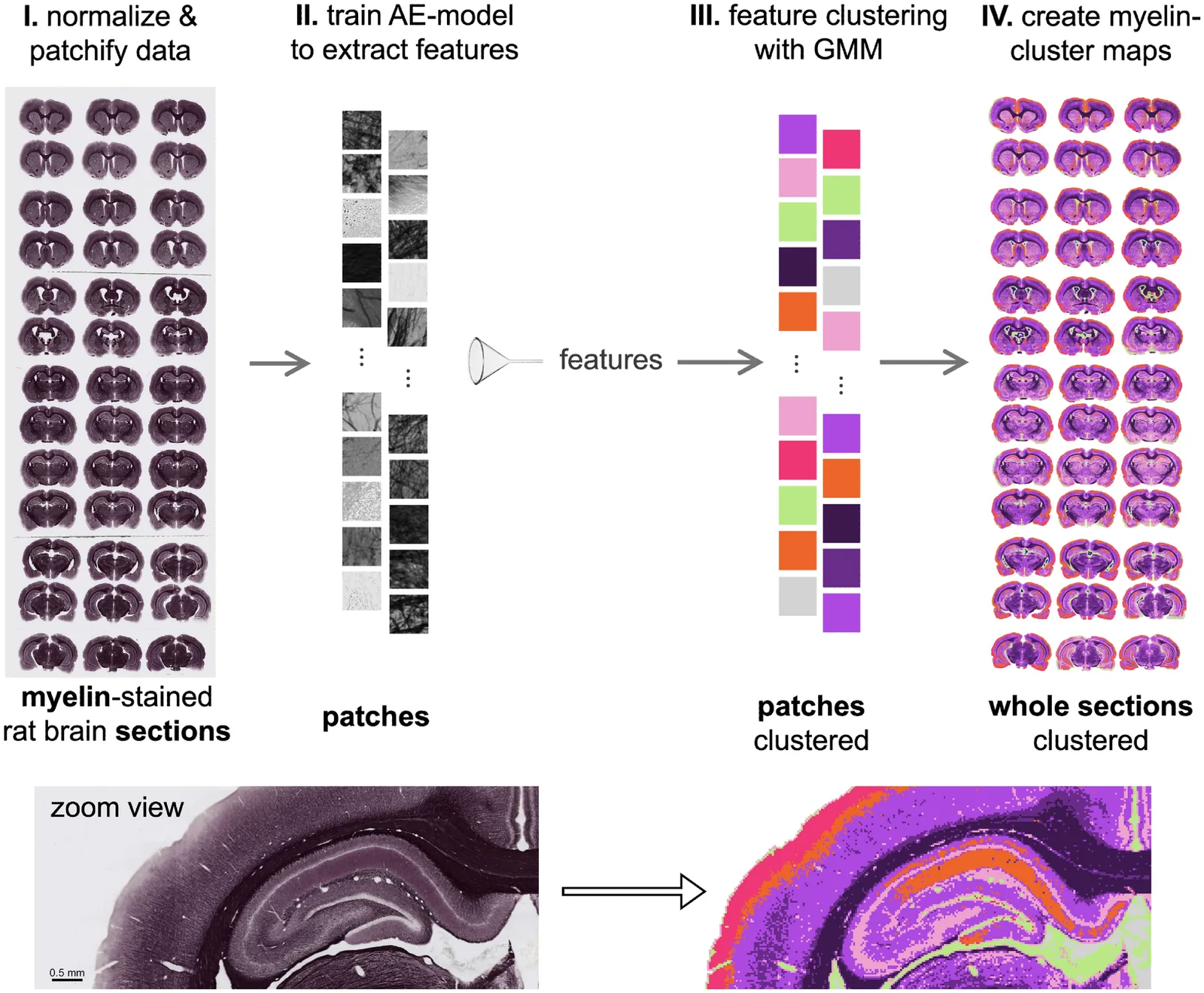
Workflow for feature extraction and classification of myelin histological rat brain sections. First, the myelin-stained rat brain sections are intensity-normalized and divided into non-overlapping patches at two scales: 128 × 128 pixels (model128) or 256 × 256 pixels (model256). A mid-coronal brain section is ~40,000 × 60,000 pixels, producing over 146,000 non-overlapping 128 × 128 pixel patches or over 36,000 256 × 256 pixel patches. Second, patches are used to train AE-models for feature extraction (256 features per patch). Third, the extracted features are clustered using Gaussian mixture models (GMM). Last, cluster assignments are projected back onto the tissue sections to create myelin-cluster maps. At the *bottom*, a zoom view of original tissue and corresponding cluster assignments

We developed a computational workflow (Fig. 1) to quantify how patterns of myelinated axons are distributed across brain tissue sections. To capture these spatial patterns, we systematically extracted non-overlapping square tissue patches at two scales (128 × 128 pixels and 256 × 256 pixels) from intensity-normalized myelin-stained rat brain sections.

We then trained convolutional AEs to compress each patch into 256 latent features and compared their performance against PCA and DINOv3 for dimensionality reduction. AEs and PCA models were trained on patches from 9 rat brains (4 sham-operated and 5 with mild traumatic brain injury (mTBI); 420 sections in total) and validated on 4 independent rat brains (2 sham-operated and 2 with mTBI; in total 194 sections). DINOv3 was used out of the box, as a feature extractor without fine-tuning. Results presented below are all from the validation set.

To automatically separate tissue organization patterns, we clustered the extracted features using Gaussian mixture models (GMM) (McLachlan and Peel 2000), identifying distinct tissue clusters based on myelin properties.

### AEs preserve structural detail better than PCA despite higher reconstruction error

**Fig. 2:**
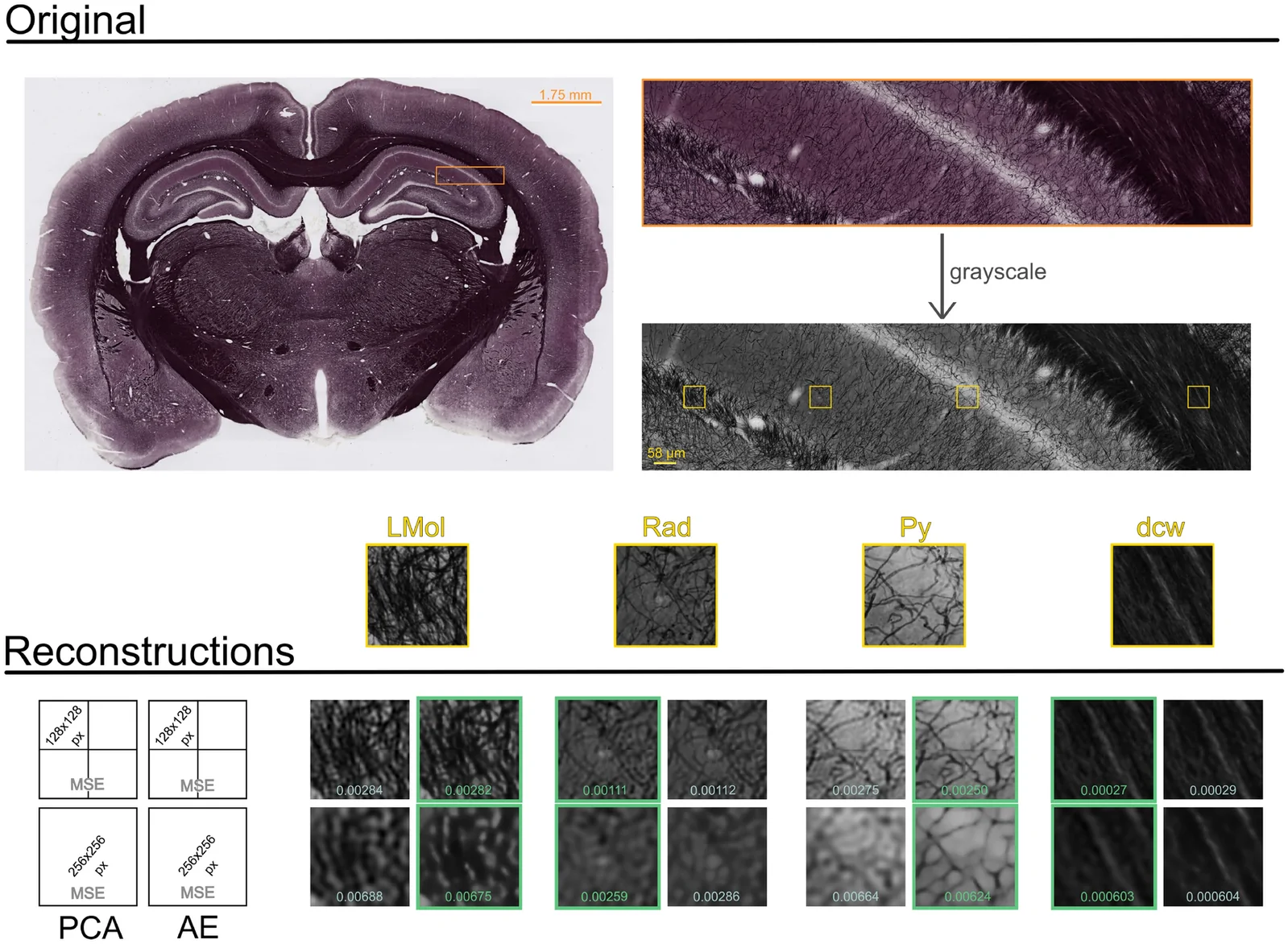
Original and reconstructed myelin-stained tissue patches. Representative mid-coronal section of a myelin-stained section of a sham-operated rat. Four tissue windows (*58× 58* µm, *256 × 256* pixels) were reconstructed using four models: PCA-based and AE-based of both model128 and model256. Mean square error (MSE) values are included for each window, with *green borders* highlighting the better-performing model in each pair. Anatomical regions are: LMol (lacunosum molecular layer of the hippocampus), Rad (radiatum layer of the hippocampus), Py (pyramidal cell layer of the hippocampus) and dcw (deep cerebral white matter) (Paxinos and Watson 2014)

We reconstructed tissue patches from the features extracted by PCA and AEs to assess feature quality for each method (Fig. 2). Reconstructions were not performed for DINOv3 features, as this would have required training a dedicated pixel-wise decoder on our data, which was beyond the scope of this work. AE model reconstructions preserved thin axonal structures and tissue texture more accurately than PCA. PCA reconstructions smoothed fine details but maintained overall tissue organization. When comparing input patch size, model128 reconstructed fine axonal features better in both PCA and AE. In contrast, model256 captured broader structural patterns but lost small-scale detail due to its higher compression ratio. AE reconstructions were more faithful in preserving myelinated axon patterns.

**Table 1:** Reconstruction quality comparison for model128

model128	MSE ($\times 10^{-3}$) *↓ *		SSIM *↑ *	
	PCA	AE	PCA	AE
sham 1	2.160	2.243	0.6914	0.6909
sham 2	1.923	1.954	0.6863	0.6894
mTBI 1	1.972	2.042	0.6930	0.6918
mTBI 2	3.414	3.411	0.6568	0.6666
mean	**2**.**367**	2.412	0.6819	**0**.**6847**
std	0.705	0.677	0.0170	0.0121
Cohen’s d	*-0.065*		*-0.189*	

MSE and SSIM values for PCA and AE across four validation animals. MSE values are scaled by $10^{-3}$ for readability. Lower MSE and higher SSIM indicate better reconstruction. Cohen’s d shows negligible effect sizes, with PCA slightly lower in MSE and AE slightly higher in SSIM. Bold values indicate the better-performing method for each metric

**Table 2:** Reconstruction quality comparison for model256

model256	MSE ($\times 10^{-3}$) *↓ *		SSIM *↑ *	
	PCA	AE	PCA	AE
sham 1	4.909	5.120	0.5179	0.5228
sham 2	4.253	4.412	0.5196	0.5257
mTBI 1	4.002	4.183	0.5411	0.5442
mTBI 2	6.146	6.360	0.4948	0.5016
mean	**4**.**828**	5.019	0.5183	**0**.**5236**
std	0.959	0.979	0.0189	0.0174
Cohen’s d	*-0.197*		*-0.287*	

MSE and SSIM values for PCA and AE across four validation animals. MSE values are scaled by $10^{-3}$ for readability. Lower MSE and higher SSIM indicate better reconstruction. Cohen’s d shows small effect sizes favoring PCA for MSE and AE for SSIM. Bold values indicate the better-performing method for each metric

We assessed the reconstructions quantitatively through mean square error (MSE) and structural similarity index measure (Wang et al. 2004) (SSIM). Individual patches showed highly similar MSE values between PCA and AEs, with each method outperforming the other on different tissue regions (Fig. 2).

For MSE averaged across animals’ sections, PCA achieved slightly lower values than AE for both patch sizes (Tables 1 and 2). For model128, the difference was negligible (Cohen’s d = −0.065). For model256, the effect remained small (Cohen’s d = −0.197).

For SSIM, the pattern reversed, favoring AE with modest effect sizes. For model128, AE achieved higher SSIM than PCA, though the effect size was small (Cohen’s d = −0.189). For model256, the advantage for AE was more pronounced (Cohen’s d = −0.287). These findings suggest that while PCA achieved slightly better pixel-wise reconstruction accuracy, AE better preserved structural features, particularly when processing larger patches that capture more complex spatial patterns.

PCA models were trained in less than 3 CPU days, while AEs training required 6 GPU days for model128 and 9 GPU days for model256. AEs training used early stopping, halting when the validation error stopped improving for 10 consecutive iterations. Model128 achieved lower training and validation errors than model256 (Supplementary Figure 8).

### PCA, DINOv3 and AE features enable identification of anatomically distinct tissue clusters

We performed unsupervised clustering using Gaussian mixture models (GMM) on the extracted features from PCA, DINOv3 and AE models to evaluate whether each model’s features classified the tissue into biologically meaningful groups. We selected three, nine, and 21 as the number of clusters based on Bayesian Information Criterion (BIC) (Steele and Raftery 2010) analysis (see Supplementary Figure 9, 10 and 11). This analysis showed an elbow at nine clusters with continued improvement with increasing number of clusters, suggesting different levels of tissue organization across multiple scales.

**Fig. 3:**
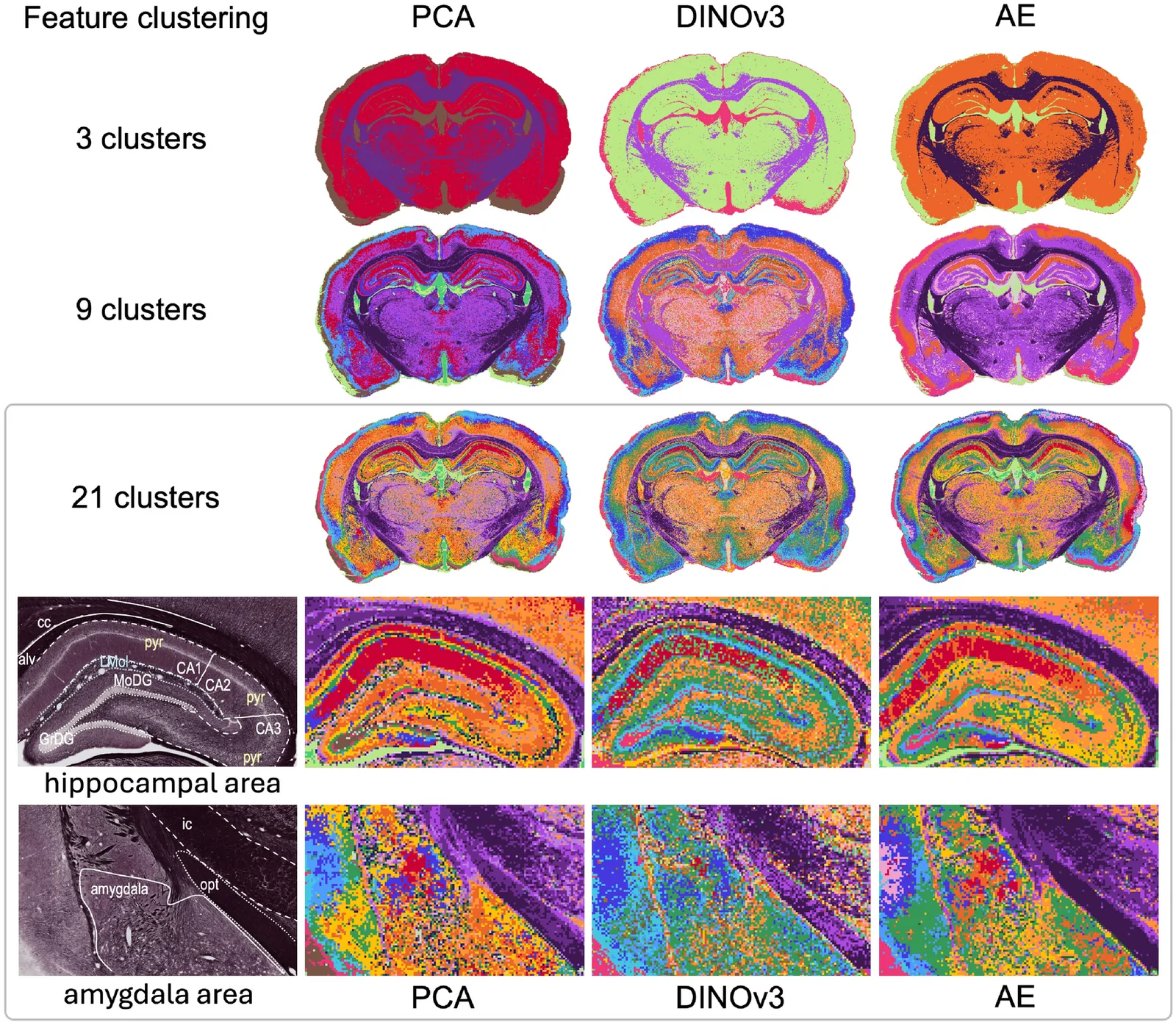
Comparison of PCA, DINOv3 and AE feature clustering. *Upper panels* show cluster assignments at three, nine and 21 clusters for PCA (*left*), DINOv3 (*middle*) and AE (*right*) features of the same section. *Lower panel* shows magnified hippocampal and amygdala areas at 21 clusters: the original myelin staining (*bottom left*), then PCA clustering, DINOv3 clustering and AE clustering. Cluster colors were assigned at 21 clusters and matched for lower cluster numbers via symmetric KL divergence. Anatomical abbreviations in alphabetical order: *alv* alveus of the hippocampus, *CA1* field CA1 of the hippocampus, *CA2* field CA2 of the hippocampus, *CA3* field CA3 of the hippocampus, *cc* corpus callosum, *GrDG* granule cell layer of the dentate gyrus, *ic* internal capsule, *LMol* lacunosum moleculare layer of the hippocampus, *MoDG* molecular layer of the dentate gyrus, *opt* optic tract, *Py* pyramidal cell layer of the hippocampus (Paxinos and Watson 2014)

Figure 3 shows the clustering results from model128. When the number of clusters was three, all three models revealed similar broad anatomical divisions: highly myelinated areas (white matter, thalamic, and hippocampal areas); grey matter areas (thalamic, hippocampal, and cortical areas); and low intensity areas (borders with low staining intensity, ventricles, and the granule cell layer of the dentate gyrus). When the number of clusters was nine (Fig. 3 second row), methods resolved in more refined tissue structures including white matter, hippocampal subfields, and cortical layers. Differences in spatial organization, particularly in the cortical layers and hippocampal region, began to emerge among the feature extraction approaches. When the number of clusters was 21 (Fig. 3 third row), each method's features enabled detailed tissue characterization but with differences in fine-grained regional grouping. White matter was mainly separated into clusters ,  and  by all methods reflecting variation in myelin density. In the cortex, AE-based features achieved clearer layer separation with clusters ,  and , whereas PCA- and DINOv3-derived clustering showed less clear stratification in this region.

To visualize relationships across the three, nine and 21 cluster groups, we manually assigned colors to the 21 clusters. We then propagated these colors to the 9-cluster and 3-cluster maps based on feature-space proximity, measured with the symmetric KL divergence. For example, the highly myelinated areas identified at nine clusters are subdivided into more specific subclusters at 21 clusters, reflecting finer distinctions in myelin density and axonal organisation rather than a reorganisation of the clustering solution. Interestingly, this hierarchical refinement was most consistent in AE-derived features: AE consistently assigned white matter to the same cluster  across all cluster numbers, while PCA and DINOv3 assigned white matter to different clusters depending on the total number of clusters. This suggested that AE captured white matter as a more compact, intrinsically distinct property.

A closer examination of the hippocampus and dentate gyrus at 21 clusters (lower panels of Fig. 3) showed that the AE-feature-map had a clear cluster gradient of regions CA1, CA2 and CA3 of the hippocampus (with clusters , , ), whereas the PCA- and DINOv3-feature maps showed a less gradual change. All methods distinguished the lacunosum molecular layer, molecular layer of the dentate gyrus, and the granule cell layer of the dentate. PCA and DINOv3 delineated the pyramidal cell layer throughout the CA3 region more effectively than the AE-map. In contrast, zoomed views of the amygdala and optic nerve highlighted advantages of the AE representation in feature clustering. Specifically, clusters , ,  clearly demarcated the optic nerve and internal capsule in the AE-map, whereas these structures were less distinctly separated in the PCA- and DINOv3-maps. The AE-map also yielded more compact and anatomically coherent delineations of amygdalar subregions compared with the other methods.

Overall, AE-feature clustering showed greater stability across clusterings with different number of clusters and sharper anatomical boundaries than PCA- and DINO-feature clustering. Subsequent results will therefore focus on AE-features.

### Centroids and probability maps enable anatomical interpretation of tissue clusters

**Fig. 4:**
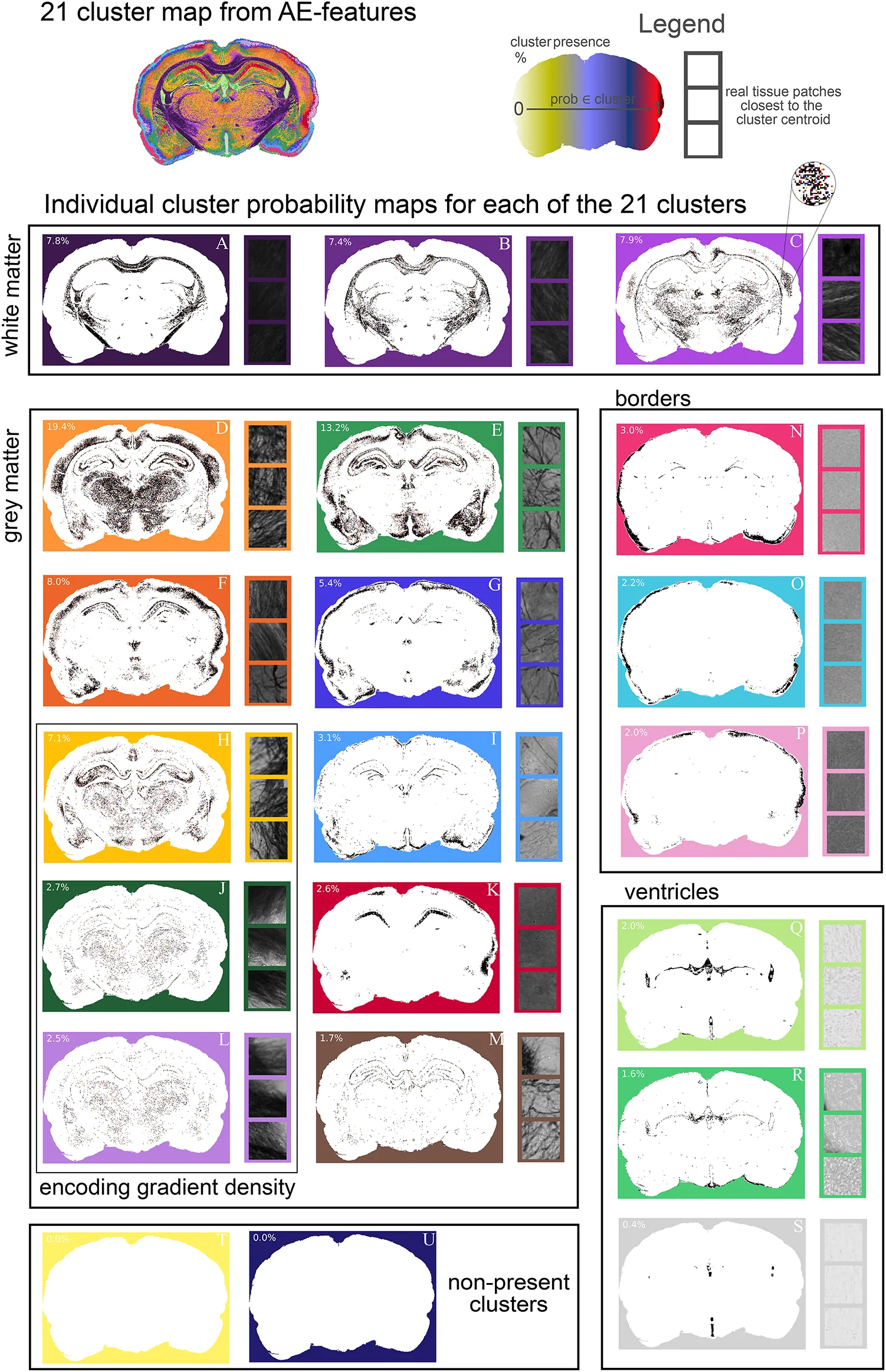
Probability maps of a single section. On the *top left*, overview of the 21-cluster map from AE-features from one section. On *top right*, the legend shows the color scale of the probability maps. The individual cluster probability maps are grouped in: white matter, grey matter, borders, ventricles and non-present clusters; each with the percentage of its presence in the tissue section, a letter for easier reference to the cluster, and three actual tissue patches (*128 × 128* pixels) closest to that cluster centroid

We sought to understand what tissue properties each cluster represents and its spatial distribution. Fig. 4 shows the probability maps of the 21-cluster map from the AE-features of model128, and the tissue patches whose latent space representation is closest to the cluster centroid. We found that in the mid-coronal section depicted in the figure, the white matter comprised 23.1% of total tissue content across the clusters , , . Cluster  captured densely packed myelinated axons; cluster  captured highly myelinated, oriented axons with inter-axonal spacing; cluster  represented highly myelinated borders of major tracts (corpus callosum, internal and external capsules, optic nerves) with greater axonal separation.

Grey matter fell into 10 clusters. Cluster  (19.4%) captured regions with abundant myelinated axons with varied orientations (with both aligned and crossing patterns) and visible inter-axonal spacing. Cluster  (13.2%) showed sparse, randomly oriented myelinated axons. Cluster  (8%) captured myelinated regions with loosely packed axons. Interestingly, clusters , ,  captured myelin-density gradients at boundaries between high and low myelin content.

Clusters ,  and  captured the section borders without myelinated axons but with varying tissue staining intensities. Three additional clusters, ,  and  captured ventricles and some tissue borders with staining artifacts. In this particular section, two of the 21 clusters were absent.

We observed no systematic differences between the left and right sides of the tissue section during clustering. This means that the resulting clusters were rotation-invariant although this was not explicitly enforced by the modeling scheme.

### Clustering identifies pathology-associated changes in white matter and myelin organization

To assess whether unsupervised clustering could capture tissue damage induced by mTBI, we analyzed myelin-stained sections from two sham-operated controls and two mTBI-injured animals (mTBI1 with subtle tissue damage and mTBI2 with evident tissue damage). We performed GMM on features extracted from patches across all four animals, enabling identification of common tissue properties and pathology-specific variations.

**Fig. 5:**
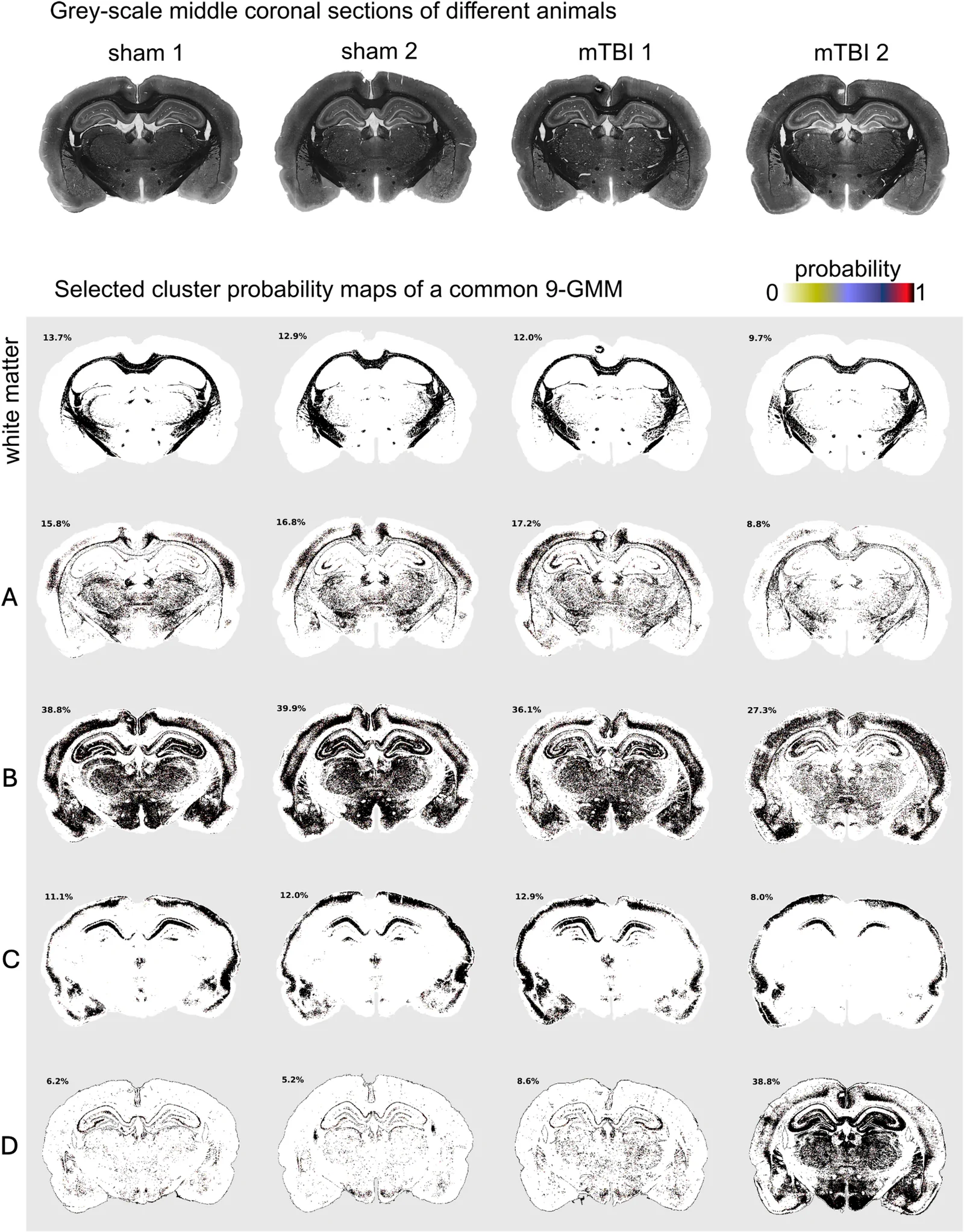
Representative cluster probability maps of a common 9-component GMM of four animals. Four representative coronal sections from four different animals (two sham and two after mild traumatic brain injury (mTBI)) and selected cluster maps from a common 9-component GMM

We present five representative clusters from the 9-component GMM model (probability maps shown in Fig. 5). The first cluster delineated intact white matter, showing consistent presence in sham controls (~13%) and mTBI1 (12%) but notably reduced in mTBI2 (9.7%), with striking asymmetry on the left (injured) side. Clusters A and B represented myelinated axons within grey matter regions at varying densities. In shams and mTBI1, clusters A and B represented >16 and >36% of tissue respectively; in contrast, these decreased to 8.8 and 27.3% in mTBI2. Most notably, cluster D captured border regions and hippocampal areas, with low presence in sham controls (~5–6%) and modest elevation in mTBI1 (8.6%) but dramatic increase in mTBI2 (38.8%). This shift strongly suggested that cluster D specifically captured pathological tissue associated with mTBI.

## Discussion

Brain histology requires scalable methods that move beyond labor-intensive manual annotations and predefined features sets (Azizi et al. 2022; Huang et al. 2025). We proposed an unsupervised workflow that automatically quantifies spatial patterns formed by myelinated axons, enabling distinction of tissue organization and detection of pathological changes without requiring manual labeling of tissue classes or pathological features.

In the context of previous literature, our findings extend the use of unsupervised deep learning in histology. While prior studies have applied similar methods to non-brain tissues, such as H&E-stained colon (Rmouque et al. 2022), pancreatic (Rumman et al. 2023), or breast and lung tissues (Sheikh et al. 2022), myelin-stained brain sections present unique challenges. White matter contains densely packed, overlapping myelinated axons with varying orientations, while grey matter exhibits lower but highly variable axonal density with diverse crossing patterns. In regions like the cortex, these patterns transition gradually without clear boundaries. Successfully characterizing such tissue requires methods that preserve fine axonal details while capturing broader organizational features.

Our workflow extracts features from patches of myelin-stained rat brain sections and clusters them to identify distinct tissue patterns. To evaluate feature extraction performance, we reconstructed image patches from the latent representations learned by PCA and AEs, and compared clustering results across all three methods. Patch reconstruction demonstrated that the AE preserved fine structural details of myelinated axonal tissue more effectively than PCA, while also revealing that reconstruction error (MSE) did not reflect the preservation of biologically meaningful features. Clustering the extracted features using GMM recovered known neuroanatomical structures with all three methods. At three clusters, AE-, PCA-, and DINOv3-derived features delineated major tissue compartments (white matter, grey matter, borders and ventricles). Increasing the number of clusters to nine and 21 revealed progressively finer anatomical organization, including white matter subtypes, hippocampal subfields, and cortical layers. Among the evaluated approaches, AE-derived features provided the most anatomically coherent representations, yielding more stable cluster assignments across resolutions and sharper anatomical boundaries, particularly within the cortex, hippocampus, and amygdala. DINOv3 features effectively captured large-scale structural organization but were less successful in resolving subtle variations in myelin density when applied without domain-specific adaptation. This limitation likely reflects the discrepancy between the diverse natural image datasets used to train DINOv3 and the highly specialized texture patterns present in myelin-stained tissue, which are unlikely to be represented in its training data. Overall, these findings suggest that task-specific training on myelin-stained tissue enhances feature sensitivity to the fine-grained microstructural complexity of myeloarchitecture, resulting in more biologically informative tissue representations.

When AE-features from sham and mTBI animals were clustered together, the workflow identified pathology-associated signatures without prior injury information. A cluster that represented a small fraction of tissue in sham animals (5–6%) increased to 38.8% in one injured animal, capturing subtle tissue damage that may include axonal injury, inflammation, or glial changes. This suggests the ability to detect heterogeneous injury patterns across brain regions.

Recent work has shown that myelin organization patterns carry biological significance. Myeloarchitectonic variations relate to functional network organization (Royer et al. 2020), axonal morphological changes at the micrometer scale occur in neurological disorders (Abdollahzadeh et al. 2025; Coronado-Leija et al. 2024), and brain lipid distributions align with functional anatomy (Fusar Bassini et al. 2025). Our workflow demonstrates that unsupervised deep learning can automatically identify biologically meaningful tissue patterns and detect pathology without manual labeling, capturing pattern-level variation in axon density, orientations, and fiber crossings that classical intensity-based or fiber orientation approaches alone do not resolve, and enabling scalable annotation-free approaches (Aggarwal et al. 2025; Cooper et al. 2023).

This study has several limitations. The models were trained on a single experimental batch comprising nine rat brains and validated using four independent animals from the same batch. Although the dataset is substantial by conventional histological analysis in terms of the number of sections analyzed, validation based on only four animals remains limited. Importantly, the appropriate unit of validation is the animal rather than the individual section, as sections derived from the same animal share biological variability and therefore cannot be considered independent observations. Furthermore, model generalizability across different staining protocols, laboratories, or experimental batches was not assessed. Consequently, the findings should be interpreted with appropriate caution and require replication in larger and more diverse cohorts. Future studies should also determine the minimum training set size required to achieve stable feature representations and systematically evaluate how performance varies as a function of the number of animals included in model training. Although histogram matching reduced intensity-related variability, more advanced stain normalization approaches, originally developed for other histological stainings such as H&E, may further improve model robustness if adapted to myelin staining protocols (Huang et al. 2025). The pathology-associated cluster observed in mTBI animals is particularly intriguing; however, its biological significance remains unclear and warrants further investigation. Integration of histological and molecular markers could help elucidate its cellular and biochemical composition and determine whether it reflects inflammation, gliosis, axonal injury, or a combination of these processes. Finally, the present analysis treated histological sections as two-dimensional images, despite the inherently three-dimensional organization of myelinated tissue. Future developments in automated 3D histology, including pipelines that combine block-face imaging with whole-brain analyses (Vandenberghe et al. 2016), could extend the proposed framework to volumetric characterization of tissue architecture. Additional future directions include integrating cluster-level analyses with fiber-orientation mapping and deriving explicit morphometric descriptors from within clusters, thereby enabling a more comprehensive characterization of myeloarchitecture.

In this work, we tested whether unsupervised deep learning could be used to identify meaningful patterns in myelin-stained rat brain tissue sections without any labels. Our results show that convolutional AEs capture fine axonal organisation details more effectively than both linear decomposition and generic visual representations applied out of the box, and that clustering these features recovers known neuroanatomical structures while also detecting pathology-associated features. These findings confirm that task-specific nonlinear unsupervised methods can overcome the limitations of traditional, object-centric histology workflows, capturing pattern-level variation in myeloarchitecture that classical metrics alone do not capture. By establishing a framework for label-free characterisation of tissue architecture, our work addresses a gap in computational neuropathology and provides a foundation for future studies extending this approach to other staining methods, tissue types, or disease models.

## Materials and methods

### Animals and histology

We used photomicrographs of 13 adult male Sprague-Dawley rats (10 weeks old, 300–450 g, Harlan Netherlands B.V) from a previous study (Molina et al. 2020). Seven animals underwent mild traumatic brain injury (mTBI) induced using the lateral fluid percussion (LFP) injury model (Kharatishvili et al. 2006). Briefly, under anesthesia, a 5 mm craniectomy was performed on the convexity of the left skull. An LFP injury induced a mild brain injury (0.93 ± 0.22 atm). Six other animals (shams) underwent the same surgical procedure except for the injury, serving as sham-operated controls. All experimental procedures were approved by the Animal Ethics Committee of the Southern Finland Provincial Government and conducted in accordance with the guidelines set by the European Union Directives 2010/63/EU.

**Fig. 6:**
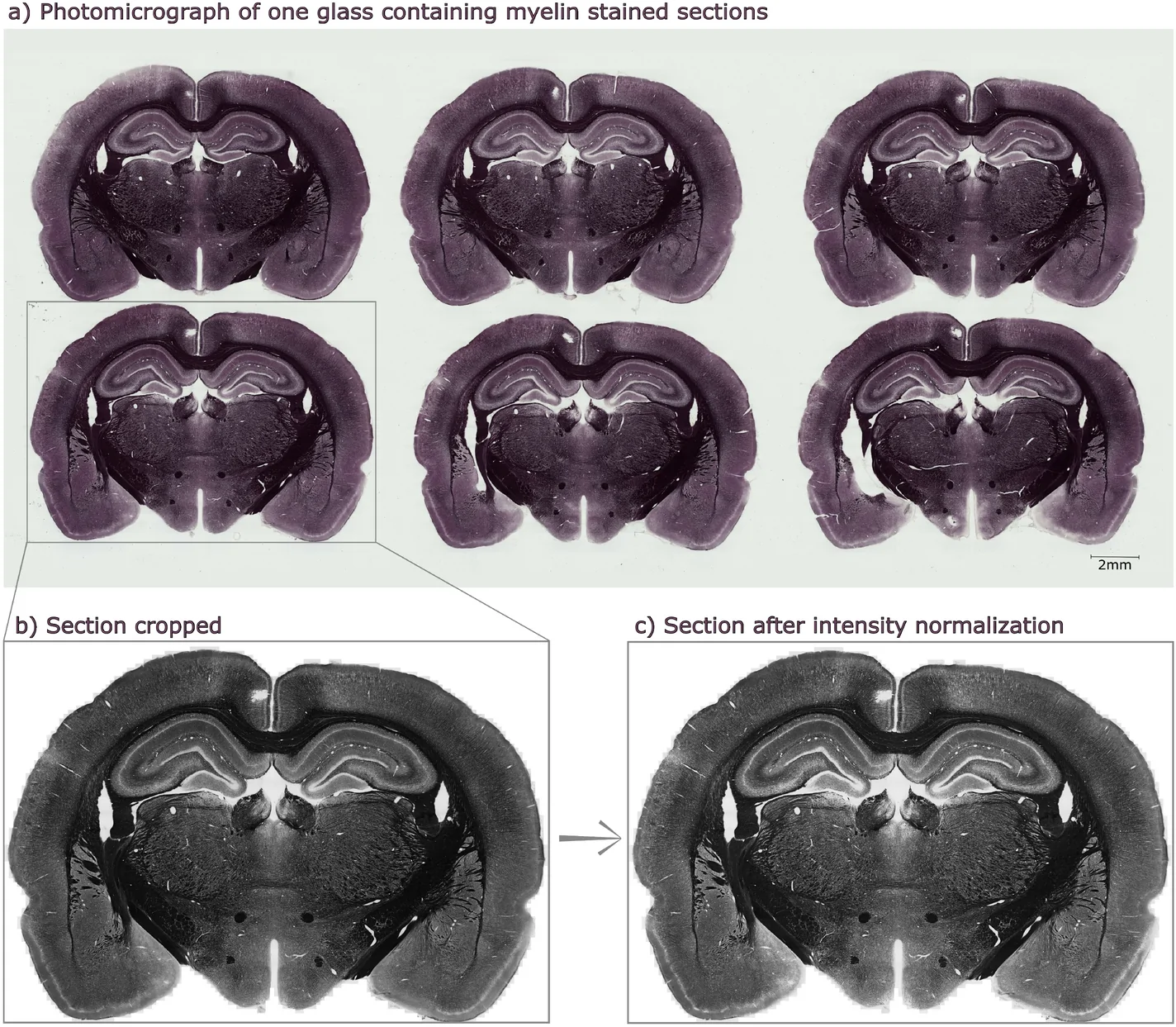
Preprocessing: **a** photomicrograph of a glass containing six myelin-stained sections; **b** one section after cropping; **c** same section after intensity normalization

Thirty-five days after surgery, all rats were anesthetized and transcardially perfused 4% paraformaldehyde (more details in Molina et al. 2020). After perfusion, the brains were removed from the skull, postfixed, and then placed in a cryoprotective solution and frozen to store at −70 °C until sectioning. All brains were sectioned in the coronal plane (30 *μ *m, 1-in-5 series), using a sliding microtome. The data used in this study corresponds to one series stained with gold chloride for myelin (Laitinen et al. 2010; Molina et al. 2020) to assess myeloarchitecture. After staining, high-resolution photomicrographs of the myelin-stained sections were acquired using a Hamamatsu scanner, NanoZoomerXR. The images were acquired at 40×, with a resolution of 226 nm/px. Each photomicrograph corresponds to one microscope slide that contains typically six brain sections (Fig. 6a) with the same staining.

### Image preprocessing

Image preprocessing consisted of first separating the photomicrographs of glasses into individual image files containing only one section; and second correcting for intensity staining variations across the sections. We preprocessed photomicrographs of sections +2.28 mm to −7.56 mm from bregma (Paxinos and Watson 2014). This selection resulted in a total of 592 photomicrographs.

We converted the photomicrographs to grayscale, downsampled them by a factor of $2^{6}$, and created a mask for each section. The masks served to define the coordinates of a bounding box containing each section. These coordinates were then rescaled to the original image size and used to crop each section (Fig. 6b), which was saved in grayscale at the original resolution. As a result, we obtained individual images for each section.

We performed histogram matching (Gonzalez and Woods 2008) to correct for staining-intensity variations across sections, using a main reference section per brain to define the target intensity distribution. An example of intensity normalization is shown in Fig. 6c. For each brain, background pixels were removed using the corresponding section masks prior to normalization. To select a reference section, we identified the five central slices of each brain series. These central slices were chosen from an intermediate position in the rostrocaudal axis, where the myelin density and distribution showed regional variability across brain regions. Across animals, these five central slices consistently captured the same anatomical level, providing a reproducible selection window. From these five candidates, one section was selected manually as the normalization reference based on staining quality: sections exhibiting an intensity representative of an optimally well-stained myelin section were preferred. This manual step reflects the known intensity variability of myelin staining even across consecutive sections from the same animal.

Once the reference section per animal was selected, we performed intensity normalization in three steps. (1) Within each brain, we performed iterative histogram matching in two directions (rostral and caudal), starting from the middle reference section. The reference section was histogram matched with its immediate neighbors—one section rostrally and one section caudally. The corrected sections then served as references for their respective next neighbors, with the process continuing iteratively in both rostral and caudal directions until all sections of the brain were processed. (2) We normalized across the different brains by selecting one brain from a sham-operated rat as the global reference and histogram matched the reference sections of all other brains to it. (3) We repeated the first step using the updated reference sections from step two, propagating the cross-brain normalization throughout all sections of the brain.

### Feature extraction

Of the 13 animals included in this study, 2 sham-operated and 2 mTBI animals animals were reserved for independent evaluation as a test set. The 2 mTBI animals in this group represented different pathological profiles, one with clear structural damage and the other with subtle injury. From the remaining 9 animals, one mTBI brain was randomly selected as the validation set (for the AE, not used in PCA). The remaining 8 brains (4 sham-operated and 4 mTBI) were used for training AE and PCA models. To minimize animal-specific bias, sections from all animals in the training set were shuffled before training AE and PCA. For both PCA and AE, we trained two separate models with different input patch sizes (*128 × 128* pixels and *256 × 256* pixels), extracting 256 features from each patch. The *128 × 128* pixel size was chosen as the smallest patch that preserved sufficient histological information, while the patches of *256 × 256* pixels enabled comparison of model performance across spatial scales. The background patches were excluded from training. The number of training patches per section depended on the input patch size. For input patch size of *128× 128* pixels, we extracted 102,400 non-overlapping patches per section, and for input patch size of *256× 256* pixels we worked with 25,600 non-overlapping patches per section. Unlike PCA and AE, DINOv3 was used as a pretrained feature extractor without any fine-tuning or retraining on our dataset.

#### Principal component analysis (PCA)

Given the large size of the dataset, we applied Incremental PCA (Ross et al. 2008), which processes data in batches to enable memory-efficient decomposition. It was implemented using scikit-learn IncrementalPCA, with a batch size of 1024 samples and 256 components.

#### DINOv3

We used DINOv3 ViT-B/16 (Siméoni et al. 2025) as a pretrained feature extractor, without any fine-tuning on our dataset. To match the model’s required input dimensions, patches were resized from their original size (128 × 128 or 256 × 256 pixels) to 224 × 224 pixels prior to feature extraction. Although DINOv3 supports RGB input, patches were kept as grayscale to maintain consistency with the PCA and AE pipelines, with the single channel replicated across all three input channels. Input patches were normalized using standard ImageNet statistics (mean = [0.485, 0.456, 0.406], std = [0.229, 0.224, 0.225]). DINOv3 encoded each patch into a 768-dimensional feature vector.

#### Autoencoders (AE)

**Fig. 7:**
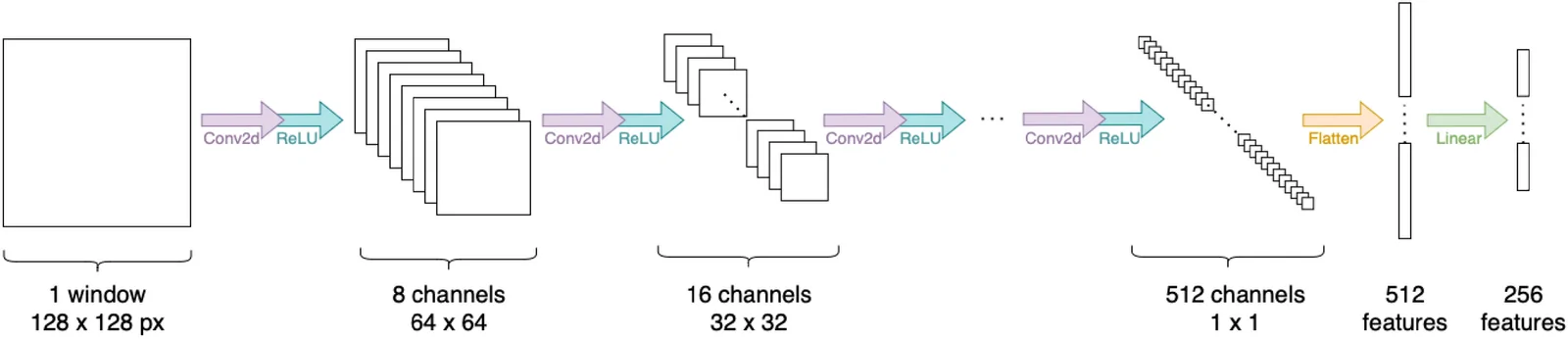
Diagram of the encoder architecture with input window size of *128 × 128* px and 256 features extracted

We used a convolutional AE architecture (Goodfellow et al. 2016) (Fig. 7) to extract features from tissue patches. Regardless of the input patch size, the encoder’s first convolutional layer extracts 8 channels from the input patch while decreasing by half the patch size. The consecutive convolutional layers duplicate the number of channels, while decreasing by half the patch size. We used as many layers as necessary to reach a patch size of *1 × 1*. Then, we flattened the vector and applied a linear layer to get the desired feature dimension. We applied batch normalization after each convolutional layer, followed by the ReLU activation function. The decoder was a mirror of the encoder architecture, except for a final sigmoid layer to ensure bounded output values in the range [0, 1]. All convolutional encoder and decoder layers used a kernel size of 3 and stride of 2.

We trained the model using MSE loss function and Adam optimizer with a learning rate of $10^{-3}$. The model was trained in batches of 64 windows. To prevent overfitting, we implemented early stopping with a patience of 10, meaning that training was halted if validation loss failed to improve for 10 consecutive evaluations. Validation loss was assessed periodically throughout each epoch, after processing every 1/8 of the training data. The best-performing state of the model was saved before patience counting began.

The dataset was processed dynamically, with patches extracted on demand rather than stored in memory. We combined Pytorch DataLoader with a window extraction function to handle this process. The training pipeline was implemented in Pytorch and executed on GPU resources from Puhti supercomputer at CSC – IT Center for Science, Finland. A single NVIDIA V100 GPU was allocated for training, along with 80 GB of memory.

### Feature analysis

#### Reconstruction quality assessment

To quantify reconstruction quality, we employed two complementary metrics: MSE and SSIM (Wang et al. 2004).

MSE quantifies pixel-wise intensity differences between original and reconstructed images. For images *I* (original) and *R* (reconstructed), the MSE of the pair (*I*, *R*) was computed only on tissue pixels (i.e. where the mask > 0) as follows:

$$\begin{aligned} \text {MSE} = \frac{1}{N}\sum _{k=1}^{N}(I_k - R_k)^2, \end{aligned}$$

where *N* is the total number of tissue pixels within the mask.

SSIM measures perceptual similarity by comparing local patterns of luminance, contrast, and structure, providing complementary information to MSE about the preservation of spatial features. For images *I* and *R* normalized to [0,1], SSIM is defined as:

$$\begin{aligned} \text {SSIM}(I,R) = \frac{(2\mu _I \mu _R + C_1)(2\sigma _{IR} + C_2)}{(\mu _I^2 + \mu _R^2 + C_1)(\sigma _I^2 + \sigma _R^2 + C_2)}, \end{aligned}$$

where $\mu _I$, $\mu _R$ are mean intensities; $\sigma _I$, $\sigma _R$ are the standard deviations; $\sigma _{IR}$ is the covariance; and $C_1 = (k_1 L)^2$, $C_2 = (k_2 L)^2$ are stabilization constants with $k_1=0.01$, $k_2=0.03$, and *L* the dynamic range of pixel values (Wang et al. 2004). Given the large dimensions of the whole-brain images, we computed SSIM on non-overlapping patches of 2048 × 2048 pixels, which were then averaged across all tissue-containing patches per section (defined as patches with more than 10% of tissue based on brain mask). This approach maintains the sensitivity of SSIM on local structural features while enabling efficient computation.

The values shown in Tables 1 and 2 represent the mean of the MSE and SSIM values per animal obtained from its sections.

To assess the practical significance of reconstruction quality differences between PCA and AE, we computed Cohen’s d (Cohen 1988), which standardizes the difference between group means by their pooled standard deviation:

$$\begin{aligned} d = \frac{\bar{x}_1 - \bar{x}_2}{\sqrt{(s_1^2 + s_2^2)/2}}, \end{aligned}$$

where $\bar{x}_1$, $\bar{x}_2$ are group means and $s_1$, $s_2$ are standard deviations. This metric quantifies how many standard deviations separate the two methods. Effect sizes are conventionally interpreted as small (*|d| ≈ 0.2*), medium (*|d| ≈ 0.5*), or large (*|d| ≈ 0.8*) (Cohen 1988).

#### Unsupervised clustering

GMM is a probabilistic clustering approach that models data as a weighted sum of multiple Gaussian distributions (McLachlan and Peel et al. 2000). Each cluster is characterized by a mean and covariance matrix. We fit GMM using the Expectation-Maximization (EM) algorithm, which iteratively estimates cluster parameters and assigns probabilistic memberships to data points (Dempster et al. 1977).

After parameter exploration, we configured GMM with spherical covariance (independent variance per component) and covariance regularization of $10^{-3}$. Model parameters were initialized using k-means clustering and a regularization parameter of $10^{-3}$. For each animal, we randomly selected 100,000 tissue patches, extracted 256-dimensional features via PCA or AE encoders, and fit GMM on these feature representations.

We evaluated cluster numbers from 3 to 27 (in steps of 3, Supplementary Figure 9, Supplementary Figure 10 and Supplementary Figure 11) using BIC (Steele and Raftery 2010) to balance model fit with complexity. We selected three numbers of clusters for detailed analysis: 3 clusters to represent major tissue types; 9 clusters, corresponding to the elbow in the BIC curve where improvement plateaued across all animals; and 21 clusters to reveal finer tissue details while maintaining interpretability.

*Cluster matching via symmetric KL divergence*

To enable visual comparison across different numbers of clusters, we manually assigned colors to the 21-cluster solution, then sequentially propagated these colors to solutions with fewer clusters (18 down to 3, in steps of 3). For each propagation step, we computed the symmetric Kullback–Leibler (KL) divergence between all pairs of cluster distributions, modeled as spherical Gaussian distributions (multivariate normal with spherical covariance). We used the Hungarian algorithm to find the optimal one-to-one cluster assignment that minimized the total symmetric KL divergence, with each cluster inheriting the color of its optimal match.

Assuming two distributions $P \sim \mathcal {N}(\mu _0,\,\Sigma _0)$, $Q \sim \mathcal {N}(\mu _1,\,\Sigma _1)$ of dimension *k*, the symmetric KL divergence from *Q* to *P* is defined as

$$ D_{KL}(P \Vert Q) = \dfrac{1}{2} \left[ tr (\Sigma _1^{-1}\Sigma _0)-k+ (\mu _1 - \mu _0)^T \Sigma _1^{-1}(\mu _1 -\mu _0) + \ln {\dfrac{|\Sigma _1|}{|\Sigma _0|}} \right] . $$

Let *I* be identity matrix of rank *k*. For spherical multivariate normal distributions, $\Sigma _j = \sigma _j^2I$ and therefore $|\Sigma _j| = \sigma _j^{2k}$ and $\Sigma _j^{-1}=\dfrac{1}{\sigma _j^2}I$ for *j = 0,1*. Therefore, symmetrized KL divergence is:

$$ S_{D_{KL}} = D_{KL}(P \Vert Q) + D_{KL}(Q \Vert P) = \dfrac{1}{2}\left[ k\left( \dfrac{\sigma ^2_0}{\sigma ^2_1}+\dfrac{\sigma ^2_1}{\sigma ^2_0}\right) -2k + \dfrac{\Vert \mu _1-\mu _0\Vert ^2}{\sigma ^2_1}+\dfrac{\Vert \mu _0-\mu _1\Vert ^2}{\sigma ^2_0} \right] .$$

*Probability maps and real tissue cluster representatives*

For each tissue patch, the GMM model outputs probabilities for all clusters. From each tissue section, we generated spatial probability maps for every cluster, visualizing both the distribution and the confidence of the assignments. We also identified feature vectors nearest to each cluster centroid and mapped them back to the original patches, providing interpretable examples of what each cluster represents in the image space.

*Cross-animal clustering*

To assess the consistency and generalizability of cluster organization across animals, we performed clustering on a combined dataset. From each of the 4 animals used for evaluation, we randomly selected 25,000 tissue patches (excluding background), yielding a total of 100,000 patches from the 4 different animals. We applied the GMM on this dataset, and compared the cluster distribution across animals.

## Supplementary Information

Below is the link to the electronic supplementary material.

Supplementary file 1 (pdf 14446 KB)

## Data Availability

Our source code is available on GitHub (https://github.com/UEF-Multiscale-Imaging/AE-and-GMM-myelin-stained-tissue). Data used in this paper will be shared by the lead contact, Alejandra Sierra (alejandra.sierralopez@uef.fi) upon request. Requests are typically answered within 2 weeks.
