# Supplementary material for: Autoencoders for unsupervised analysis of rat myeloarchitecture
Source: Brain Struct Funct. 2026 Sep 26;231(8):141. doi: 10.1007/s00429-026-03166-w (PMC13616018; doi:10.1007/s00429-026-03166-w)
Supplement: Supplementary file 1 [file 429_2026_3166_MOESM1_ESM.pdf]

5 Supplementary Figures

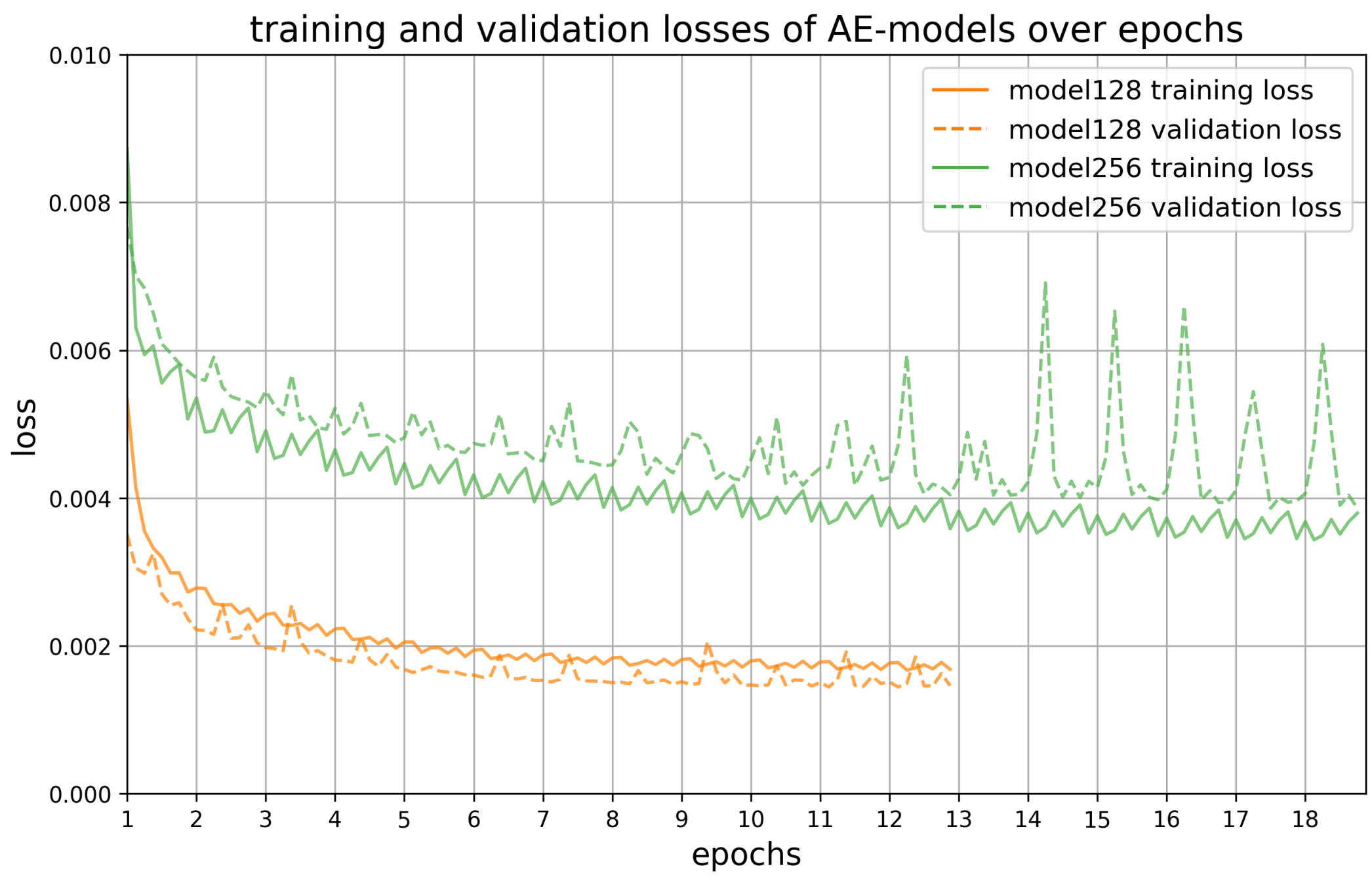

Figure 8: Training and validation loss curves for AE model128 and model256. Model128 shows stable convergence with training and validation losses remaining closely aligned. Model256 exhibits intermittent spikes in validation loss during the final third of training, suggesting potential instability when learning features from larger patches.

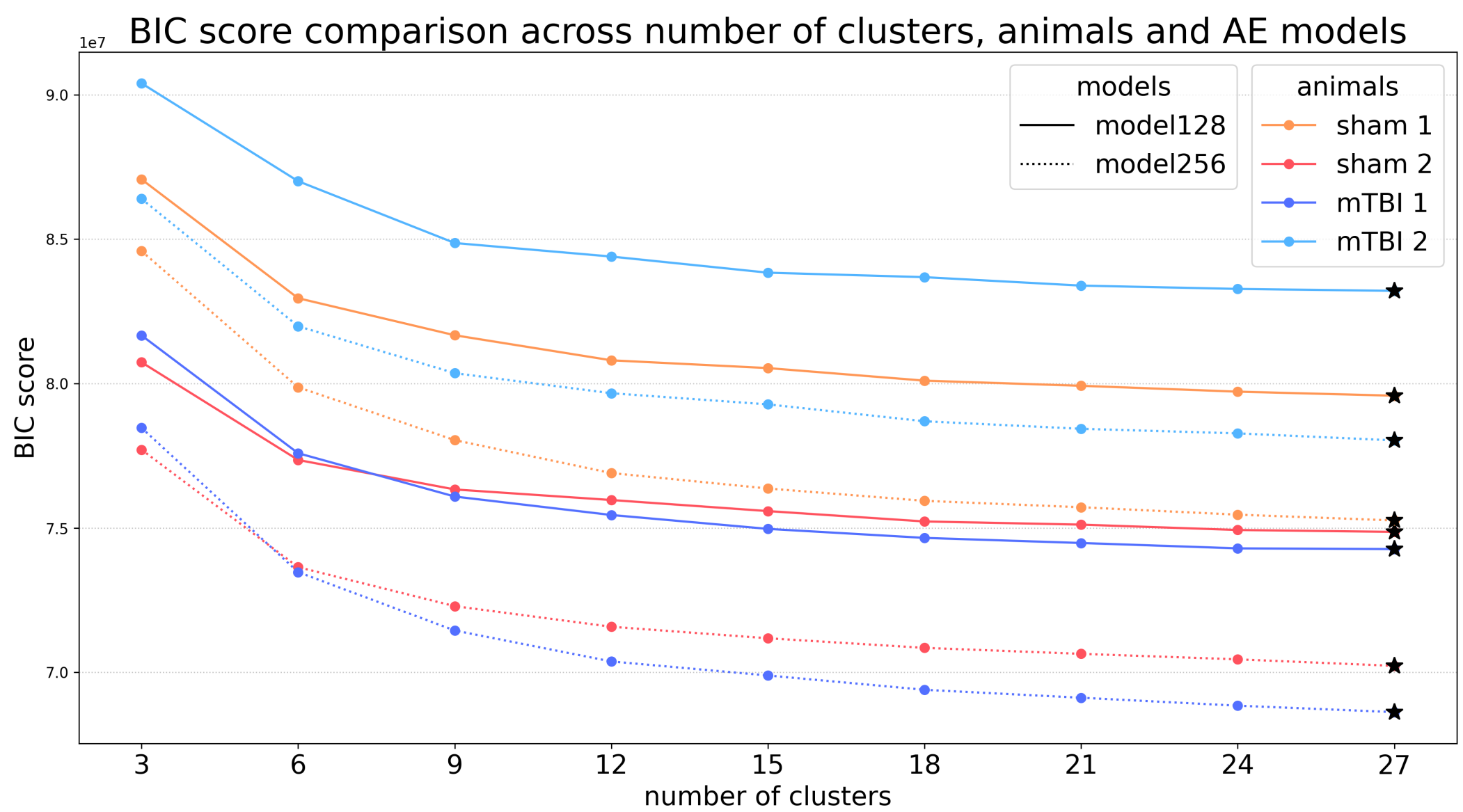

Figure 9: BIC scores from clustering AE-features. Regardless of the model or animal, the greater the number of clusters, the lower the BIC score, achieving the lowest (star symbol) at 27 clusters in this cluster range. The elbow is considered at 9 clusters, when the BIC score starts to stabilize.

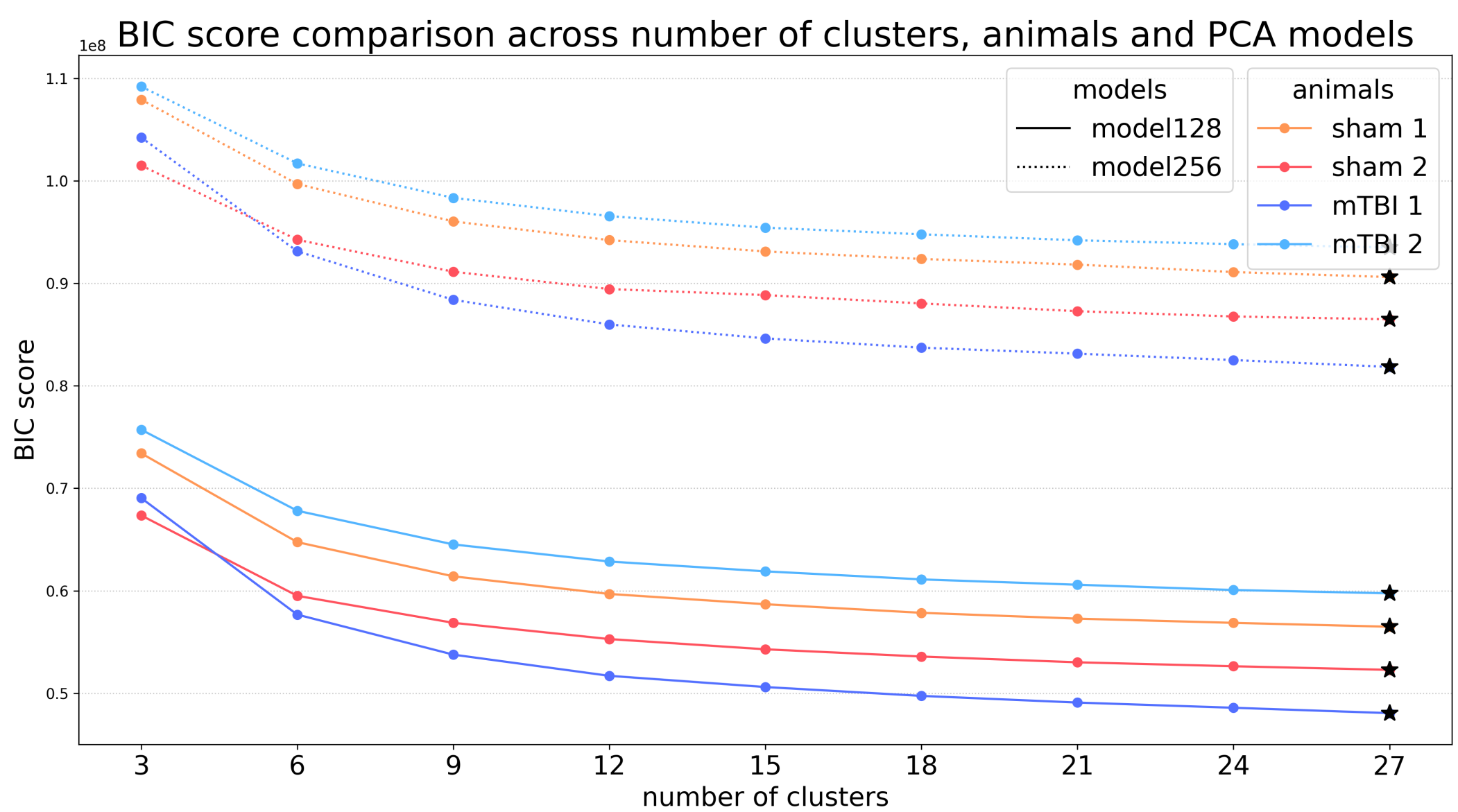

Figure 10: BIC scores from clustering PCA-features Regardless of the model or animal, the greater the number of clusters, the lower the BIC score, achieving the lowest (star symbol) at 27 clusters in this cluster range. The elbow is considered at 9 clusters, when the BIC score starts to stabilize.

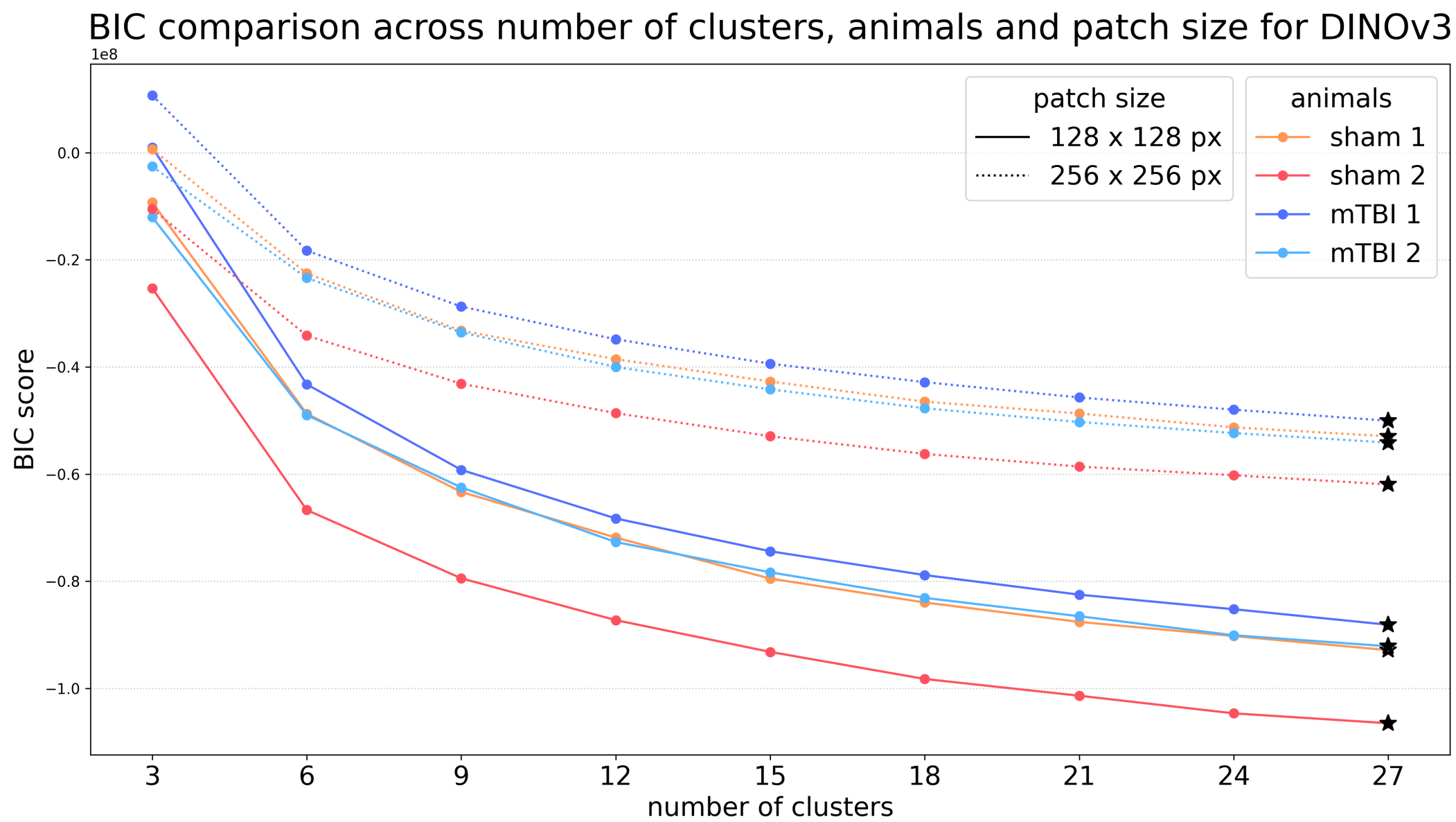

Figure 11: BIC scores from clustering DINOv3-features Regardless of the patch size or animal, the greater the number of clusters, the lower the BIC score, achieving the lowest (star symbol) at 27 clusters in this cluster range. The elbow is considered at 9 clusters, when the BIC score starts to stabilize.

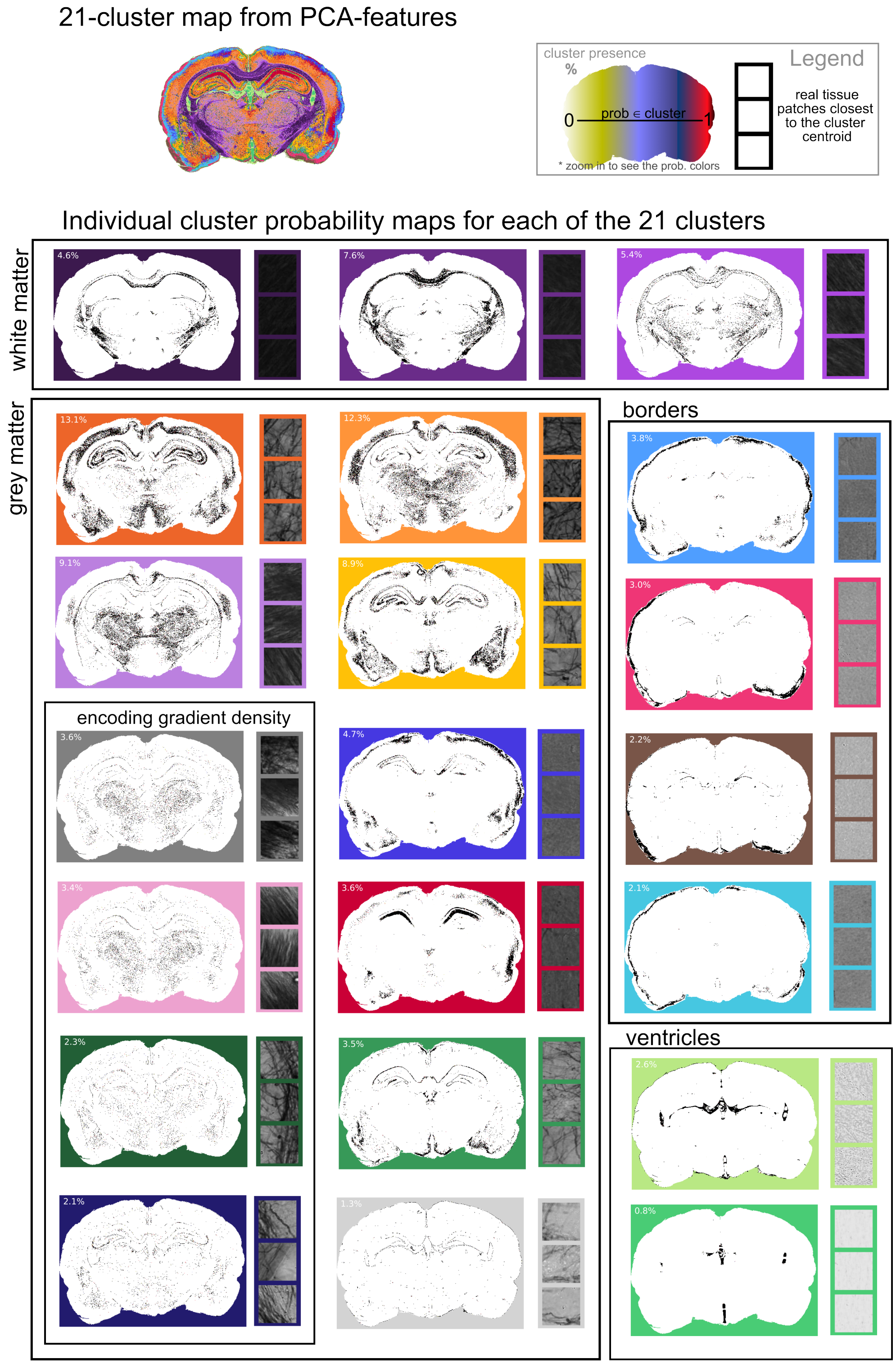

Figure 12: Probability maps of a single section. On the top left, overview of the 21-cluster map from PCA-features from one section. On top right, the legend describing the color scale of the probability maps. Then, the individual cluster probability maps grouped in: white matter, grey matter, borders and ventricles. Each probability map shows a percentage that indicates its presence in the tissue section, as well as three real tissue patches (128x128 pixels) closest to that cluster centroid.

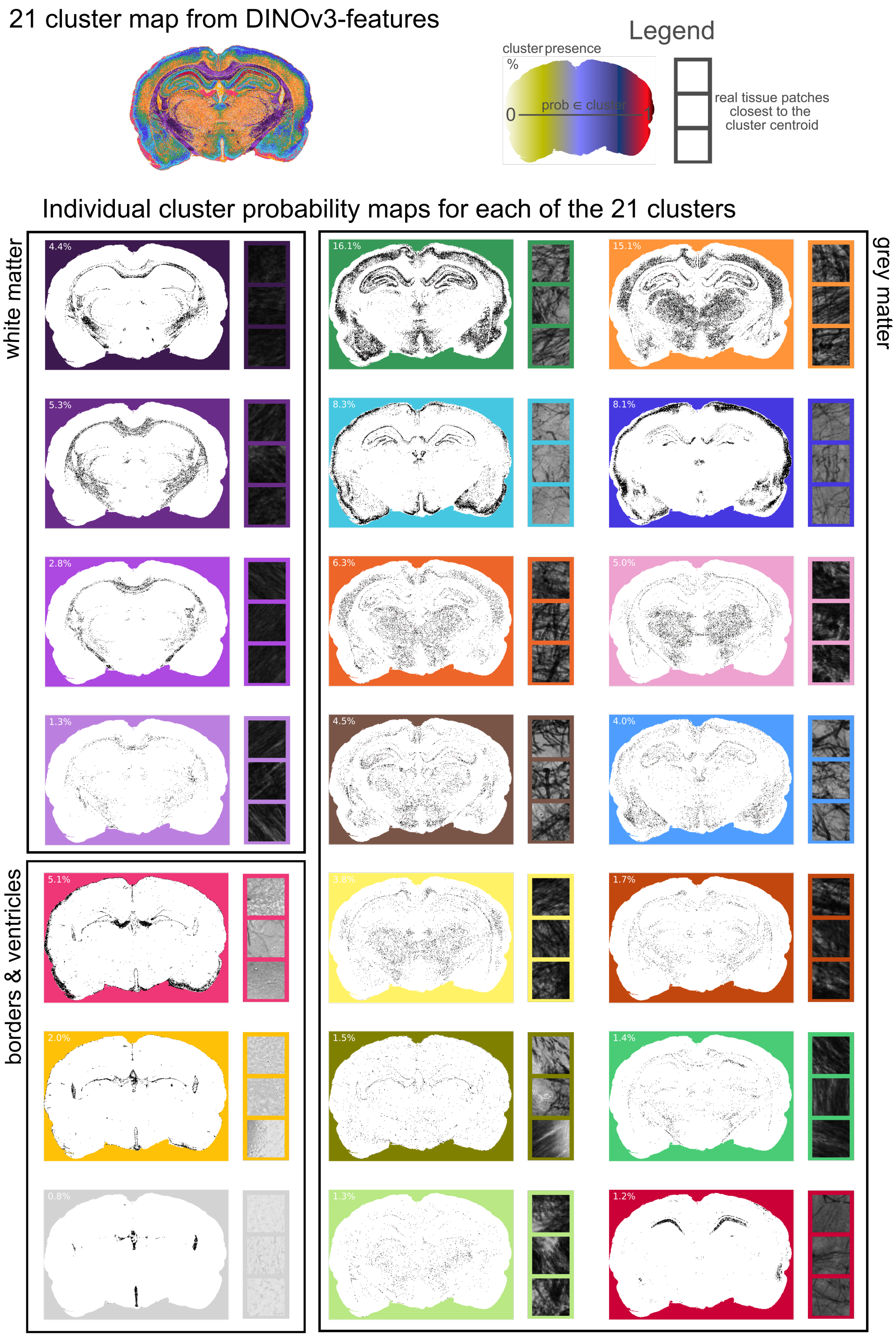

Figure 13: Probability maps of a single section. On the top left, overview of the 21-cluster map from DINOv3-features from one section. On top right, the legend describing the color scale of the probability maps. Then, the individual cluster probability maps grouped in: white matter, grey matter, borders and ventricles. Each probability map shows a percentage that indicates its presence in the tissue section, as well as three real tissue patches (128x128 pixels) closest to that cluster centroid.
